# Organic Supercapacitors as the Next Generation Energy Storage Device: Emergence, Opportunity, and Challenges

**DOI:** 10.1002/cphc.202200567

**Published:** 2022-11-03

**Authors:** Sudipta Biswas, Ananya Chowdhury

**Affiliations:** ^1^ Department of Chemistry Ben Gurion University of the Negev Beer Sheva, Southern District Israel; ^2^ Department of Chemistry Indian Institution of Technology Bombay Mumbai Maharashtra India

**Keywords:** energy storage, green energy, organic pseudocapacitor, organic supercapacitor, supercapacitor

## Abstract

Harnessing new materials for developing high‐energy storage devices set off research in the field of organic supercapacitors. Various attractive properties like high energy density, lower device weight, excellent cycling stability, and impressive pseudocapacitive nature make organic supercapacitors suitable candidates for high‐end storage device applications. This review highlights the overall progress and future of organic supercapacitors. Sustainable energy production and storage depend on low cost, large supercapacitor packs with high energy density. Organic supercapacitors with high pseudocapacitance, lightweight form factor, and higher device potential are alternatives to other energy storage devices. There are many recent ongoing research works that focus on organic electrolytes along with the material aspect of organic supercapacitors. This review summarizes the current research status and the chemistry behind the storage mechanism in organic supercapacitors to overcome the challenges and achieve superior performance for future opportunities.

## Graphical abstract

**Harnessing new materials** for developing high‐energy supercapacitors set off research in the field of organic supercapacitors. These are novel kinds with supercapacitors with attractive properties like lower device weight but high energy density, rapid cycling stability, and most importantly very high pseudocapacitance. This review highlights the overall progress and future of such next‐generation supercapacitors and device components.

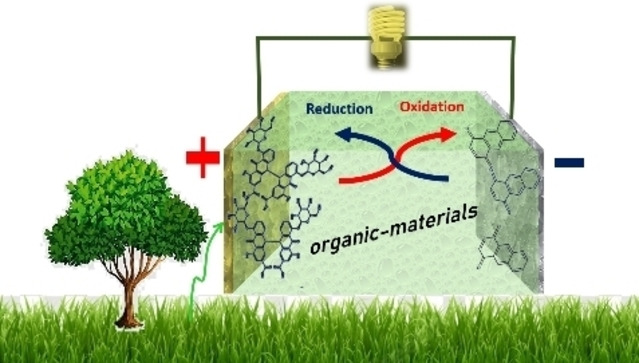

## 1 Introduction

The growing worldwide energy requirement is evolving as a great challenge considering the gap between demand, generation, supply, and storage of excess energy for future use.^[1]^ Till now the main source of the world‘s energy depends on fossil fuels which cause huge degradation to the environment.[^2^, ^3^, ^4^, ^5^] So, the cleaner and greener way to reduce the burden on the environment is to move towards renewable energy generation using.[^6^, ^7^, ^8^, ^9^] But if we consider the available conventional energy storage technologies, they are way behind in terms of volumetric energy density and as well as device design, to make use of renewable energy leading in energy consumption.[^10^, ^11^, ^12^, ^13^] This drives researchers to find eco‐friendly ways to store excess energy i. e. eco‐friendly energy storage devices.[^14^, ^15^, ^16^, ^17^]

Over the last decade, there is a surge in application‐based research mainly aiming at wearable electronics like biosensors or health monitoring devices, which are termed bioelectronics‐based organic materials.[^18^, ^19^, ^20^, ^21^] All the technologies evolved and flourished due to the progress in the conjugation of polymers, designing high‐conducting materials, having high strength along with easy processing, tuneability, and synthetic diversity.[^22^, ^23^] These materials have been exploited in the field of the organic field‐effect transistor (OFET), organic electrochemical transistor (OECT), etc.[^24^, ^25^, ^26^, ^27^] Important science behind such progress is the improvement of the observed current under small increments in the applied voltage. Another important direction of the organic supercapacitor is the bio‐based devices coupled with power devices made of organic components. The low weight, on‐chip feasibility of making, compatibility with the biological environment, and less manufacturing cost are some of the advantages of these devices.[^28^, ^29^]

*Now the question which arises first is that with these many advantages why this technology has not flourished yet*. The answer lies in the performance and long‐term stability of such devices. Although they are well advanced but still underperformed compared to the all‐inorganic devices. This problem is more prominent when we try to find the electron‐accepting material which will be used as a negative electrode.[^30^, ^31^] As a result, there is a gap between the proposed technology and the availability of the material in the market. But the positive news is we are getting new materials as well as technologies day by day which is making the growth of this technology faster. Discreet research in the field of electrode material, electrolytes, and the unavailability of focused reviews on such topics make these supercapacitors unnoticed.

So, in this review, we will summarize the recent progress on organic supercapacitors. Carbon electrodes can be considered organic electrode material for supercapacitors which is an old and basic form of such device. But those are not conventional organic materials that we are aiming at as the way forward to achieve high‐energy supercapacitors. So, we will try to elaborate on the application of other organic materials as electrodes, electrolytes, and separators in supercapacitors other than carbon‐based materials. Depending on the component, devices differ in working principle and performance. So, we will also try to understand the mechanism behind the working function of this device. In the end, the challenges of organic supercapacitors will also be highlighted to understand the future perspective in this field.

### 1.1 Component of a Supercapacitor

**Scheme 1:**
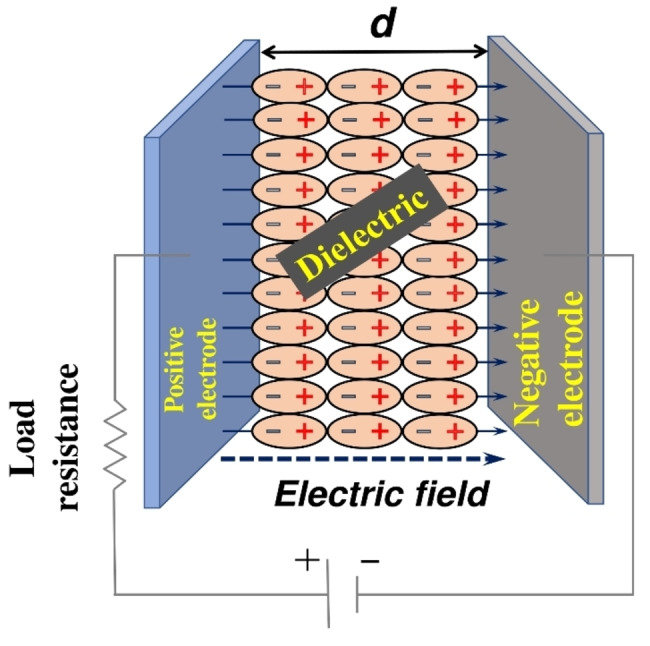
Construction of a conventional capacitor.

By changing the architecture of the device, conventional capacitors are designed to perform better, providing a huge rise in the capacitance from mF (electrolytic capacitors) and μF (dielectric capacitors) to several tens of Farad. Capacitance (C) for a conventional capacitor can be expressed as:^[32]^

$$C=\frac{\epsilon_{0}\epsilon_{r}A}{d}$$

where A is the surface area of the electrode and d is the distance between the plates. A schematic for the construction of a conventional capacitor is shown in Scheme 1. Herein, $\epsilon_{0}$ and $\epsilon_{r}$ represent the vacuum and relative permittivity, respectively. By modifying these parameters, significant improvement in performance can be obtained, and hence the terminology ‘Super’ came into the picture.

A supercapacitor mainly consists of two electrodes, an electrolyte, and a separator within an enclosure that provides the stability of the device protecting it from the external environment.^[32]^ There are many architectures for a supercapacitor, such as 3D (cylindrical, coin cell type, bulk stacked devices, etc.), 2D planner (micro supercapacitor, etc.^[33]^ Also depending on the need, the volume can be changed from small microdevices for hearing Aid, and biosensors to big packs for the car.[^34^, ^35^, ^36^]

#### 1.1.1 Electrode

The main component of a supercapacitor that stores charge to provide energy is the electrodes. The electrodes consist of a current collector, electrode material, and binder. The main concept of organic supercapacitors rises from the use of organic electrode materials. The binders that are used in electrode fabrication are mostly organic viz. Polytetrafluoroethylene (PTFE), Nafion, polyvinylidene difluoride (PVDF), etc.[^37^, ^38^, ^39^] In the electrode, the common electrode materials are carbons, transition metal oxides, transition metal sulphides, MXenes, organic aromatic compounds, polymers, etc.[^40^, ^41^, ^42^, ^43^]

#### 1.1.2 Electrolyte

**Scheme 2:**
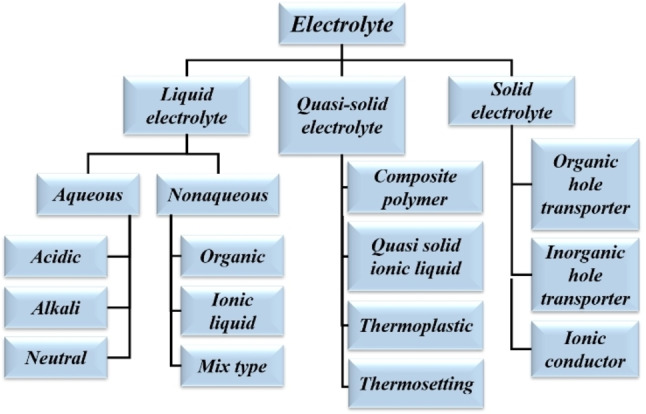
Classifications of electrolytes being used in supercapacitors.

An electrolyte is the combination of a solvent and salt, which determines the stability of the device controlling its maximum potential. Supercapacitors are developed mainly to back the Li‐ion batteries. So, the need for high potential output makes the role of electrolytes very important. The desirable properties of an electrolyte are lightweight i. e., low density, low viscosity, good electrochemical activity, high ionic conductivity, and non‐decomposition at high voltage. An electrolyte provides the electrical continuity of the electrode and also helps the redox reaction occurring in the active material present in the electrode. Popular electrolytes are acidic (H_2_SO_4_), basic (KOH, NaOH, etc.), neutral (KCl, Na_2_SO_4_, etc.), polymeric, ionic liquid, etc.[^44^, ^45^, ^46^] Among these some of them are aqueous and some are non‐aqueous. Classifications of the electrolytes are shown in Scheme 2 for both supercapacitors and DSSCs.[^47^, ^48^] A detailed discussion on organic electrolytes has been introduced in the latter section.

#### 1.1.3 Separator

A separator is another integral part of the device which separates the electrodes from being sorted in a device. However, this is ion‐permeable which maintains the circuit continuity through an electrolyte. Although the separators do not take part chemically in charge storage, they play an important role by helping the device, not to self‐discharge. The other important role of a separator is ensuring device stability at high temperatures or when there is internal heat generated within the device, by absorbing the excess heat. A good separator should have the properties like porous structure, good mechanical and chemical stability, high ionic conductive nature, lightweight, highly permeable, non‐flammable, and hydrophilic nature. Most importantly the separator should be stable in the used electrolyte as well as in the operating potential window. Additionally, a separator should be thin but in such a way that it should insulate both electrodes to avoid any possibility of a short circuit. Next, it should be a very good dielectric material and be stable when it comes close to an electrolyte. Low ionic mobility and short‐term stability some time become to cause the performance degradation of a device.

### 1.2 Differences between Supercapacitor from Batteries and Fuel Cell

Before going into the detailing of a supercapacitor lets us see which types of other energy storage devices are present in the market to compete. The bulk of the energy storage is dependent on the battery industry and a small share is taken by supercapacitors. Fuel cells come under the backup for these devices in remote or inaccessible areas with low efficiency ranging between 40–50 % on average. The batteries are mostly used for energy storage worldwide due to their high energy density and many readily available well‐established technologies associated with them. But the scope for the supercapacitors comes when there is a need for high‐powered devices. Now, to make it more industrially viable the energy density of the materials is needed to increase. The schematic of a supercapacitor is shown in Figure 3(a).

The energy density (E) of a supercapacitor can be expressed as:

$$E=\frac{1}{2}CV^{2}$$

where C is the specific capacity of the device and V is the stable voltage window with an electrolyte. In the literature, we can find that carbon‐based materials have been used extensively for fabricating high‐performing supercapacitors. But the limiting factors for such electrode materials are the surface area and porosity which can be tuned up to a certain limit. Therefore, the metal‐based oxides and sulphides became popular due to their high pseudo‐capacity contribution toward the total capacitance. To achieve high‐performing devices, many environmentally toxic transition metal‐based inorganic electrode materials are used. Also, these materials have the limitation of showing restricted redox‐active sites as in most cases the redox reactions are surface dominant. The bulk of the material does not perform such a redox reaction in most cases. To mitigate this issue, the strategy of using redox additive in the electrolyte has been adopted in recent times which is very costly and also has some limitations. A few such limitations are the reduction in the potential window and an increase in the self‐discharge. But in comparison, organic materials are evolving as self‐sustained redox‐active materials contributing toward high pseudocapacitance. The detailed mechanism is described in the later part of this review.

Another way to improve the energy density of a device is to increase the device‘s working voltage. We know that the stable window of aqueous electrolyte is 1.23 V at standard room temperature. Though there are some reports to increase the stability window much higher with the concept of water in salt, such reports are few.[^49^, ^50^, ^51^] Here comes the scope of using non‐aqueous i. e., mainly the organic electrolyte. The organic electrolytes should have good ionic conductivity and should be electrochemically inert towards other device components, with low toxicity, low cost, and a huge stable potential window compared to the aqueous ones.

### 1.3 Storage Mechanism in Supercapacitor

The performance of an energy storage device always depends on the mechanism used by the device. The storage mechanism of a supercapacitor can be classified into three categories viz. electric double‐layer capacitance, pseudocapacitance, and hybrid or battery type. Electric double‐layer capacitance utilizes the charge accumulation at the electrode‐electrolyte interface, pseudocapacitors use quick and reversible surface redox processes, whereas the hybrid type uses both mechanisms.

#### 1.3.1 Electric Double Layer Capacitance

**Scheme 3:**
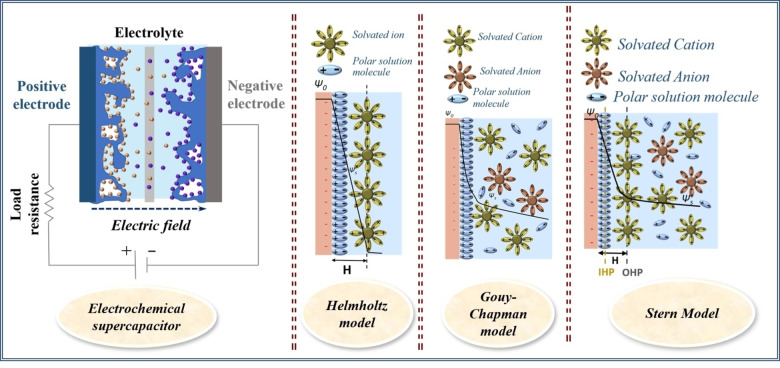
(a) Schematic for supercapacitor and charge storage mechanism for supercapacitors using (b) Helmholtz, (c) Gouy‐Chapman, and (d) Stern model.

In 1853, Helmholtz first proposed the concept of the electric double layer by studying a colloidal solution.^[52]^ This model explains the charge storage based on the adsorption and desorption of ions on the electrode/electrolyte interface. The charges would be adsorbed at the electrode surface such that the potential applied across the electrode will be dissipated linearly from the surface. A schematic for the charge storage according to the Helmholtz model is shown in Scheme 3(b).^[53]^ Gouy introduced the concept of developing an interfacial potential due to the number of charges present at the surface with an equal number of ions present in the electrolyte solution. The counter‐charge can diffuse throughout the electrolyte. Chapman developed the theory of diffused double layer considering the charged particles as point charges which follows Boltzmann distribution. This theory also has some drawbacks and cannot calculate the thickness of the diffused double layer accurately. A schematic for the charge storage according to the Gouy‐Chapman model is shown in Scheme 3(c).^[54]^ Stern modified the concept of a point charge with a specific size assigned to the charged particles. Hence the physical limitation of point charges approaching the electrode surface is removed. Further, he introduced a specifically adsorbed ion layer termed as Stern layer and beyond that another layer where one species of charge will be dominant. the charges will be evenly distributed. A schematic for the charge storage according to Stern is shown in Scheme 3(d).^[55]^

#### 1.3.2 Pseudocapacitance

**Scheme 4:**
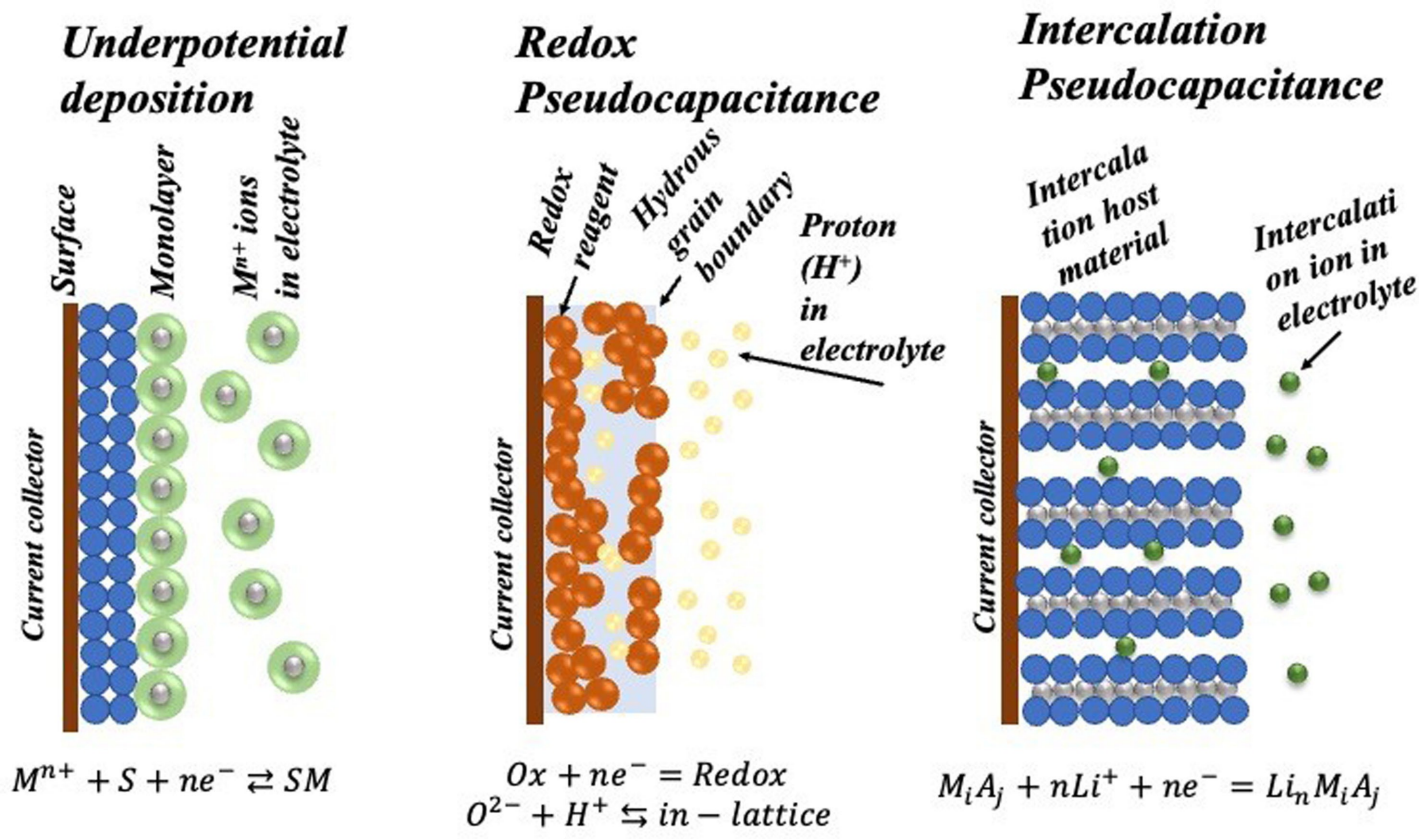
Different types of reversible redox mechanisms associated with pseudocapacitance: (a) underpotential deposition, (b) surface redox pseudocapacitance, and (c) intercalation pseudocapacitance.

In contrast to the electric double‐layer capacitance, pseudocapacitance involves the faradaic charge transfer between the electrode and electrolyte. The pseudocapacitance contribution in the total specific capacitance value is determined thermodynamically which depends on the charge accumulated (Δq) and the related potential difference (ΔU). It can be divided into two types viz. adsorption pseudocapacitance and redox pseudocapacitance. Adsorption pseudocapacitance is a two‐dimensional surface reversible process where H is adsorbed and desorbed. image of the positive and negative scan of the CV profile for an electrode. This is mainly observed in the case of 2‐dimensional materials where there is no problem associated with 3D phase change. So, depending on the material and electrolyte, if the rate constant changes, kinetic limitations may be observed. There is another reaction mechanism that should be avoided in the supercapacitor, which is underpotential deposition, depending on the strong interaction of the material and the current collector. A schematic for the different types of reversible redox mechanisms that contribute to the pseudocapacitance in a device is depicted in Scheme 4.

The other type of pseudocapacitance which is predominantly seen in most of modern supercapacitors is redox pseudocapacitance. Redox pseudocapacitance can be originated from anchored (surface‐bound) redox species or a redox reaction under a diffusion‐controlled process at an electrode‐electrolyte interface. With this, the intercalation type of pseudocapacitance is also considered in this category nowadays. For a conventional redox reaction, the peaks are generally observed at the same redox potential. On the other hand, the other kind shows a non‐mirror image CV profile where the ion intercalation and deintercalation occur at a different voltage.

#### 1.3.3 Hybrid/Battery Type Storage

The purpose of the invention of the hybrid capacitor comes from the applicability of the device. Combining two strategies mentioned earlier i. e., EDLC and pseudocapacitance with a battery‐type electrode help in achieving high energy as well as high power. Higher power is controlled by the EDLC/pseudocapacitance and the battery electrode enhances the stable potential window of the device. The new convention for a hybrid supercapacitor is where both electrodes can be EDL type electrode/redox type electrode or the second type is with a capacitor type electrode in combination with a battery type electrode in the device.

## 2 Electrode Materials for Organic Supercapacitors

With the progress in the energy storage industry especially for the supercapacitor, many electrode materials have been investigated. Many of them showed great improvement over the years. Carbon‐based electrodes were mainly used for supercapacitors due to their high surface area.[^56^, ^57^, ^58^, ^59^, ^60^, ^61^, ^62^] Though they are organic materials, in this review, the carbon structures will be excluded as the electrode material. In the late 70 s, B. E. Conway studied RuO_2_ and explored pseudocapacitance extensively.^[63]^ With the introduction of pseudocapacitance, many oxides, sulphides, and phosphates were used as electrode materials. Further materials have been tuned into different shapes and morphology with improved porosity.^[64]^ A few such materials are RuO_2_, IrO_2_, MnO_2_, NiO, V_2_O_5_, Co_3_O_4_, Fe_2_O_3_, etc.[^65^, ^66^, ^67^, ^68^, ^69^, ^70^, ^71^, ^72^, ^73^, ^74^, ^75^] Very recently the emergence of organic elements as electrode materials has been noticed.[^76^, ^77^, ^78^, ^79^, ^80^, ^81^] The concept of taking advantage of the organic chemistry in the materials for energy storage came in the 70 s. Mainly polymeric materials have been introduced as battery separators for energy storage devices.[^82^, ^83^] The main idea to realize organic electrode materials in energy storage came through the batteries. The discovery of the redox active material polyacetylene (Pac) for the battery by Shirakawa paved the way for organic energy storage.[^84^, ^85^, ^86^] But due to the lack of improvement and prosperity, it did not evolve further for supercapacitor applications. The electrochemical activity depending on both oxidation and reduction with or without doping showed improvement in the performance. This development in the field was improved with the introduction of many other conducting polymers.[^87^, ^88^, ^89^] organic electrode materials (OEM) have the advantage of high volumetric energy density which can be achieved by low volumetric mass densities. Further, this technology is improved with the development of redox‐active organic species in batteries. But till now there are very limited results on such redox active‐organic species in supercapacitors which can be nurtured to move towards green electrochemistry in supercapacitors.

Now, the relevant question which comes into the picture is what are the properties that make these electrode materials a potential candidate for such devices? Low material cost, less production cost, easy synthesis technique, and high energy density make these materials attractive to the research community forcing them to rethink using these materials for supercapacitor electrodes. Over the years polymeric materials have been utilized as a suitable candidate for such devices due to the availability of the donor and acceptor levels in the material. Another strategy to enhance the conductivity of polymers is n/p doping which also increases the redox contribution from the material. One more type of contribution comes from the conjugated polymers. Conjugated polymers are organic macromolecules that have alternative double‐ and single bonds forming the backbone of such structures. Their overlapping p‐orbitals originate the delocalized π‐electrons, resulting in high electrochemical charge storage properties. Slow ionic diffusion in the polymeric chains caused slow discharge rates in the device. All the available organic electrode materials can be divided into three subsections viz. conductive polymers, aromatic organic compounds, and metal‐organic framework. Before going into the details of the types of material used and different mechanisms employed to improve the performance, a small summary of published work in organic supercapacitors has been listed in Table 1, where mainly acidic electrolytes have been employed. In the subsequent sections, we will see the development of materials in the field of organic supercapacitors.

**Table 1:** Organic materials are used in supercapacitors.

Material	Electrolyte	Specific capacitance	Ref.
Poly(3′,4′‐ethylenedioxy‐2,2′ : 5′,2′′‐terthiophene)/ graphene nanoplatelets composite	1 M H_2_SO_4_	206 F g^−1^ at 1 A g^−1^	[90]
2D Microporous Covalent Triazine‐Based Framework	1‐Ethyl‐3‐methylimidazolium tetrafluoroborate	151.3 F g^−1^ at 1 A g^−1^	[91]
Eu‐viologen hierarchical nanosheets	0.1 M of Mn(CH_3_COO)_2_ ⋅ 4H_2_O	186.25 mF cm^−2^ at 1 mA cm^−2^	[92]
Porous graphene‐wrapped carbon foam	1 M H_2_SO_4_	129.2 F g^−1^ at 0.5 A g^−1^	[93]
Quinone	0.5 M H_2_SO_4_	216 mAh g^−1^ at a 1 C rate	[94]
Electro polymerized 2‐(thiophen‐2‐yl)furan	boron trifluoride diethyl etherate/acetonitrile	392.0 F g^−1^ at 5 A g^−1^	[95]
β‐Ketoenamine‐Linked COF	1 M H_2_SO_4_	48 F g^−1^ at 0.5 A g^−1^	[96]
carbon onions modified 4,5‐pyrenedione	1 M H_2_SO_4_	264 F g^−1^	[97]
2‐Aminoanthraquinone over Kynol Carbon Cloth	1 M H_2_SO_4_	58 mAh g^−1^ at 10 A g^−1^	[98]

### 2.1 Conducting Polymer

The most common organic electrode material is conducting polymers. These can be an efficient electric double layer‐based electrode as well as pseudocapacitance‐based electrode material. Commonly used conducting polymers are poly‐aniline (PANi), polypyrrole (Ppy), poly‐thiophene (PTh), etc. The intrinsic properties of these materials like electrical conductivity, flexibility, low material cost, and easy synthesis route make them the primary choice for the electrode of organic supercapacitors. These polymers can be synthesized by chemical or electrochemical methods. Depending on the synthesis strategy, the morphology of these materials can be altered, modulating the performance of the electrode. Along with the conductivity, the other controlling parameters for improved electrochemical performance are the porosity of the material, the surface area, and the attachment of the functional groups. There are many reports published over the years where organic polymers dominated the supercapacitor performance. The performance of these materials can be controlled by both types of energy storage mechanisms.

PANi was invented around 1886 using electro‐polymerization.^[99]^ Later Gilchrist showed electrolysis of an acid solution of aniline to achieve PANi.^[100]^ This polymer then attracted people for application in energy storage systems due to its good electrical conductivity and tunable morphology.^[100]^ If we start with PANi, it is one of the main materials that has been used as the organic electrode material or a conducting backbone for the supercapacitor electrode over the years. Diaz et al. showed appropriately the use of PANI as electrode material.^[101]^ Huang et al. also established polyaniline as the capacitor electrode material.^[102]^

Polythiophene‐based supercapacitors were introduced very early stage of the development of a supercapacitor. Laforgue et al. made supercapacitors with polythiophene (Pth) and polyparafluorophenylthiophene (PFPT) as electrode materials where they achieved around 260 F g^−1^ specific capacitance.^[103]^ Along with pure material, composites have also been explored as electrode materials. Mostly carbon nanotube is used along with the other elements as the electrode material.[^104^, ^105^] Zhang et al. used polythiophene/multiwalled carbon as the electrode material. They achieved the highest specific capacitance around 216 F g^−1^ at a current density of 1 A g^−1^.^[106]^ Qureshi et al. used an ultra‐sonication assisted strategy for polythiophene‐carbon nanotube composites for supercapacitor electrodes.^[107]^ Zhou et al. used oligo linking to attach polythiophene for having crafted composite where direct linking between the material provided good electrical conductivity and they were able to achieve 399 F g^−1^ specific capacitance at 1 A g^−1^ current.^[108]^

These improvements were not sufficient to compete with the metal oxide‐based supercapacitor. Hence other polymers and composites were also tried. One such material is polypyrrole, and it showed many advancements proving its potential as the electrode material for supercapacitors.[^109^, ^110^] With the advancement of PANI in the early 1970s, Kanazawa et al. showed the applicability of polypyrrole as an electrode material.^[111]^ They prepared highly stable, flexible films of polypyrrole with p‐type conductivities of 100 Ω^−1^ cm^−1^ by electrolytic oxidation of the appropriate pyrrole monomers. They also prepared similar films to consist of mixtures of pyrrole and N‐methyl pyrrole, having conductivities between 5×10^−3^ and 100 Ω^−1^ cm^−1^ depending upon the composition. In 1984 Feldberg showed the application of polypyrrole electrochemistry as a capacitor electrode.^[112]^

Additionally, other polymers are being tested as possible electrode materials for supercapacitor applications.[^87^, ^113^, ^114^] In 1997 Carlberg et al. utilized poly(3,4‐ethylene dioxythiophene) as electrode material in electrochemical capacitors.^[115]^ They achieved a supercapacitor cell voltage is 0.8 V with an energy density of ∼1 to 4 Wh kg^−1^. In 1998 Ferraris et al. used electroactive polymers like 3‐(4‐fluorophenyl)thiophene, 3‐(4‐cyanophenyl)thiophene, 3‐(4‐methylsulfonylphenyl)thiophene, and 3‐(3,4‐difluorophenyl)thiophene with a combination of tetramethylammonium trifluoromethanesulfonate (Me_4_NCF_3_SO_3_)/acetonitrile and/or tetraethylammonium tetrafluoroborate (Et_4_NBF_4_)/acetonitrile as electrolytes to construct devices which were able to deliver energy density of 50 Wh kg^−1^.^[116]^

### 2.2 Heteroatom Doped Organic Carbons

Carbonaceous materials with various foreign elements especially various nitrogen groups can be an option for having good electrode materials. These groups consist of graphitic N, lactam, pyridine oxide, pyrrolic N, triazine, cyano, amine, pyridinic N, etc. One of the most common heteroatom‐doped materials is graphitic carbon. There are many reports where N‐doped graphitic materials have been employed as good electrodes for supercapacitors.[^117^, ^118^, ^119^] Faisal et al. used pyridinic and graphitic nitrogen‐rich graphene for high‐performance supercapacitors.^[120]^ In this method generally, a nitrogen‐containing material is heated in presence of graphene‐based materials. They used uric acid to change the concentration of N doping which resulted in good capacitive behaviour. They achieved a 2‐electrode supercapacitor cell with a specific capacitance of 230 F g^−1^ at a current density of 1 A g^−1^ and with a remarkably high energy density of 62.6 W h kg^−1^ in an aqueous electrolyte. Elessawy et al. developed a high‐performance supercapacitor utilizing 3D nitrogen‐doped graphene structures.^[121]^ This improved the overall performance by enhancing the specific capacitance value up to 405 F g^−1^ at 1 A g^−1^. Also, the device retained performance over 87 % of the initial specific capacitance value up to 5000 cycles. There are various other reports where researchers used graphitic‐N along with inorganic materials but those will not be discussed here due to lack of scope.[^122^, ^123^, ^124^, ^125^] Next most important materials are pyridine‐based supercapacitors. Pyridine is a heterocyclic organic compound and has a chemical formula C_5_H_5_N.^[126]^ It is a benzene‐like structure with one methine group (=CH−) replaced by a nitrogen atom. Decreased electron density in the aromatic system causes fewer electrophilic substitutions in pyridine and its derivatives make the structure more stable. There are few promising reports based on pyridine and pyridine‐derived organic supercapacitors showing good perspective in the field energy storage.[^127^, ^128^, ^129^, ^130^] Troschke et al. used pyridine‐based covalent triazine frameworks (CTF) for symmetric supercapacitor electrodes and got a high specific capacitance value of 141 F g^−1^.^[127]^ Dhiman et al. invented a new class of nitrogen and phosphorus enriched pyridine bridged inorganic‐organic hybrid material for supercapacitors which showed 243 F g^−1^ capacity at 1 mV s^−1^ scan rate.^[128]^ Zhang et al. synthesized pyridine‐enriched graphene sheets (DAP‐RGOs) at mild reaction conditions from graphene oxide (GO) and 2, 6‐diaminopyridine (DAP), which resulted in a specific capacitance of 317 F g^−1^ at 0.1 A g^−1^ current density.^[130]^ Lacerda et al. used CNT‐TEPA@poly(3,4‐ethylenedioxythiophene‐co‐3‐(pyrrol‐1‐methyl)pyridine), a hybrid material to have a supercapacitor with high cycle stability.^[129]^ They achieved 52.9 F g^−1^ specific capacitance in a device at 0.25 A g^−1^ current density.

### 2.3 Conjugated Cyclic Diketones

Quinone‐based material is often used in organic supercapacitors which are known as conjugated cyclic diketones. The attractive properties of quinone‐based materials as energy storage electrodes are their high energy density, low cost, structural stability, excellent redox activity in the electrolyte, and good environmental stability.^[131]^ But there are a few drawbacks such as lower intrinsic conductivity and high self‐discharge tendency, which hinder the electrochemical applications of such materials. However, these materials possess a prime advantage which is the addition of redox activity along with the double‐layer capacitance. The application of organic quinone as electrode material was first demonstrated by Noai et al. They showed the use of poly(1,5‐diaminoanthraquinone) as electrode material for supercapacitors.^[132]^ In this work, they strategized a conducting polymer condensed with 1,4‐benzoquinone to use as electrode material which also had high conductivity of 0.3–2.0 Ω^−1^ cm^−1^. They reported high specific energy of ∼46 Wh kg^−1^. Algharaibeh et al. used anthraquinone in the negative electrode and 1,2‐dihydroxybenezene in the positive electrode using 1 M H_2_SO_4_ as the electrolyte to achieve 63 F g^−1^ specific capacitance.^[132]^ Tomai et al. used tetrachlorohydroquinone (TCHQ) cathode and dichloroanthraquinone (DCAQ) anode to fabricate a metal‐free aqueous redox capacitor via a proton rocking‐chair system using an organic‐based couple.^[133]^ This capacitor was able to show an energy density of ∼14 Wh kg^−1^ with excellent rate performance and no capacity loss even after 10,000 cycles. This proves that organic materials can be well organized to have highly stable supercapacitive performance. Katsuyama et al. very recently used chloranil and dichloroanthraquinone for stabilizing practical cells with a very high voltage window of 6 V.^[134]^ Vonlanthen et al. stabilized polyaniline‐benzoquinone‐hydroquinone as electrode materials for highly stable supercapacitors with a specific capacitance of 2646 F g^−1^ at a current density of 0.5 mA/cm^−2^.^[43]^ Suematsu et al. used supramolecular oligomer of 1,5‐diaminoanthraquinone with 4 M H_2_SO_4_ electrolyte to achieve specific capacity as high as 40–50 Ah kg^−1^.^[135]^ Zhou et al. used polyanthraquinone‐based nanostructured for very high‐performing supercapacitor.^[136]^ They succeed in achieving 650 F g^−1^ specific capacitance with more than 85 % specific capacitance retention after 1000 cycles. There are many reports where conjugated cyclic diketones have been used along with activated carbon, graphene, or CNTs to have high‐performance supercapacitors which are not discussed here.[^137^, ^138^, ^139^, ^140^, ^141^]

### 2.4 Covalent Organic Framework

Covalent organic materials are the new class of material being employed as the electrode material for supercapacitors.[^142^, ^143^, ^144^] COFs are a new class of crystalline polymeric material. They show high surface area, a good amount of porosity, and most importantly crystalline nature. These materials are linked by covalent bonding which makes them easily processible for flexible devices. Additionally, the easy synthesis strategies make these materials cost‐efficient and industry‐friendly. One of the important properties of such structures is the linking of atoms in two and three dimensions to construct extended frameworks which help the material stability as well as long‐range electrical conduction within the material. The high surface area of the material makes them good EDL electrode materials. With the addition of the functional groups to the long‐range structures, the redox incorporation can be easily done. The low density is another aspect that makes it industrially viable as a lower mass means a higher gradiometric contribution in capacitance. Though these materials have lots of advantages, still they show some drawbacks like low conductivity and relatively average capacitance for pristine material.^[145]^ The first kind of COFs has been introduced by CÔTÉ et al.^[146]^ Among the various strategies to make COFs, hydrogen bonding, introduction function groups, and compatible linking reactions have been employed. Sajjad et al. synthesized phosphine‐based porous organic polymer/rGO aerogel composites and utilized them as supercapacitor electrode materials.^[147]^ The devices made by them offered a potential window up to 1.6 V with a high energy density of 33.3 Wh kg^−1^. In other work, they showed a specific capacitance of 100 F g^−1^ at a current density of 1 A g^−1^, with an energy density of 32 Wh kg^−1^.^[148]^ DeBlase et al. invented another type of 2‐D COF with β‐ketoenamine‐linking to achieve high pseudocapacitance.^[96]^ Xu et al. constructed extensible and flexible supercapacitors from COFs to deliver high gravimetric capacitance of 249 F g^−1^.^[149]^ Haldar et al. synthesized pyridine‐rich COFs to have high‐performing solid‐state supercapacitors where electrodes can yield an excellent specific capacitance of 546 F g^−1^ at 0.5 A g^−1^.^[150]^ In another work, they showed redox‐active and hydrogen‐bonded TpOMe‐DAQ COF can be used as electrode material for supercapacitors which delivered 169 F g^−1^ specific capacitance.^[151]^ Along with the pristine COFs, we can find various reports with COF‐assisted composite for the electrode.[^151^, ^152^, ^153^, ^154^]

### 2.5 Metal‐Organic Frameworks

Metal‐organic frameworks (MOFs) cannot be included as pure organic materials as metal ions are involved in the organic framework. But for completeness of the review and to have a note for the properties in these frameworks utilized for supercapacitor electrodes which can be the notable direction for improvement in other organic electrodes. So, we have included a small mention of these materials in short. MOFs comprise metallic centers and infinite arrays of multitopic organic units. MOF structures can be multi‐orientated and can be synthesized in 0D, 1D, 2D, and 3D structures. One of the attractive properties of these materials is high crystallinity. MOF has a metal center and organic ligands with nano‐sized internal void spaces which can easily accommodate the ions.^[155]^ Though these materials are not purely organic, for the sake of completeness of this review here we will introduce MOFs and discuss their applicability as the electrodes in supercapacitors. Li et al. published an initial report on the electrode made of nanowires of conductive MOF for solid‐state supercapacitors. They reported a high specific capacitance with 204 F g^−1^ using 1 M H_2_SO_4_ as electrolyte.^[156]^ Zhang et al. synthesized hollow Ni‐MOFs to achieve 530 F g^−1^ at 0.5 A g^−1^ in 1 M LiOH aqueous solution. Sheberla et al. synthesize highly conductive porous MOF Ni_3_(HITP)_2_ with a very high areal‐specific capacitance of ∼18 μF cm^−2^.^[157]^ Yang et al. developed cobalt‐based layered MOF with nanosheet morphology.^[158]^ An electrode made of this material can deliver a maximum capacitance of 2564 F g^−1^ at a current density of 1 A g^−1^. These electrodes are also able to retain their 95.8 % capacitance after 3000 cycles. Composites with other materials like carbon and polymer also have been employed as a strategy to prepare high‐performing electrodes. Wen et al. synthesized Ni‐MOF/CNT composites for a high‐performing electrode. The electrodes were able to deliver ∼1765 F g^−1^ at a current density of 0.5 A g^−1^_._ ^[159]^ MOFs were also used in some work to synthesize porous carbon which was further used as the electrode material.[^160^, ^161^]

### 2.6 Mechanism of Charge Storage in Organic Material

**Scheme 5:**
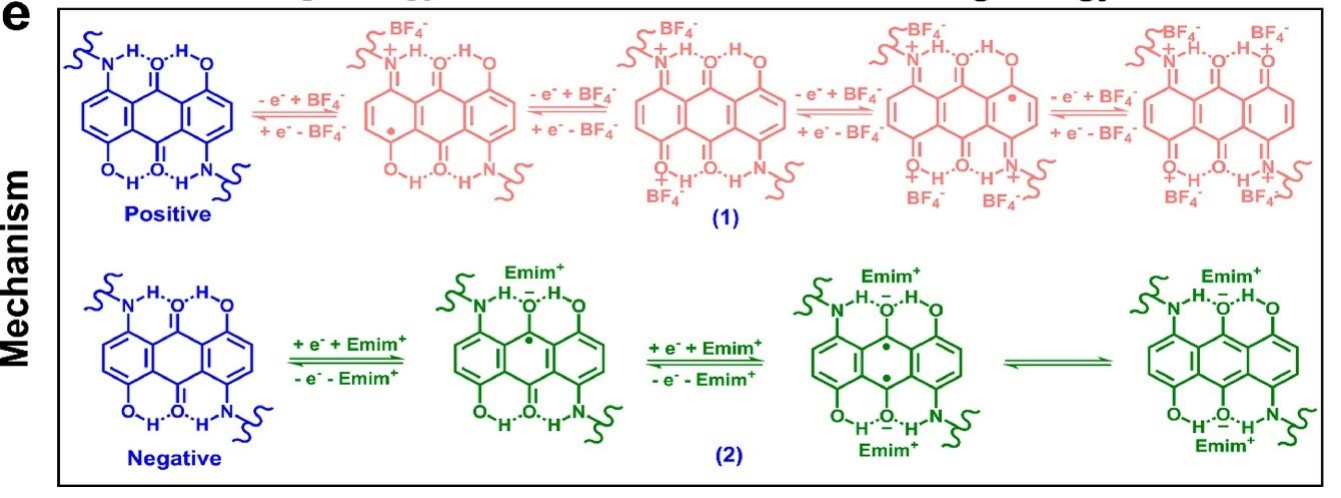
Charging mechanism of the DHAQ_3_‐NGA electrode material in EmimBF_4_ electrolyte. Reproduced from Ref. [165]. Copyright 2022, with permission from Elsevier.

Till now we have discussed the electrode materials that are employed directly in pristine, composite, or derived forms. Now let's discuss the mechanism of energy storage in those electrodes. The main mechanism behind every supercapacitor is the electrical double‐layer formation at the electrode‐electrolyte interface. Organic electrode material sometimes suffers in that aspect when there is a low surface area available for the ions to store the charges. So, other means of strategies are being employed to enhance the storage in such devices. The straightforward way to increase the energy density of such material is the introduction of reversible redox reactions between the electrolyte and electro‐active species.^[162]^ The redox‐active spices are the functional groups that can participate in the electron exchange between themselves and electrolyte ions. One of the critical influencing factors of reversible redox reactions depends on both proton‐exchanging and electron‐hopping processes within the material.[^163^, ^164^] For a detailed discussion, we can see the following mechanism addressed by Yang et al.^[165]^ They have reported a novel electrode material 1,5‐diamino‐4,8‐dihydroxyanthraquinone (DHAQ) grafted nitrogen‐doped graphene aerogel (DHAQ‐NGA) prepared by a simple hydrothermal method. As the DHAQ molecule contains −C(=O)− and −NH−/−OH groups, it can act as both the negative and positive electrode materials for a supercapacitor simultaneously. At the negative electrode, upon charging, the dual −C(=O)− groups of the DHAQ fragment in the material get successively two electrons and are reduced into dual C−O− groups. This whole process is a two‐electron transfer process. Whereas in the case of the positive electrode, upon charging, the −NH− group combined with para−OH in the DHAQ fragment loses two electrons and oxidized into −NH+− and −C=O+ groups. Therefore, two dual −NH^−^/−OH groups can lose four electrons in total. E_mim_^+^ and BF_4_^−^ served as the counterions for the negative and positive electrodes, respectively, to balance the charges.[^34^, ^35^] A possible mechanism for redox reactions occurring at the positive and negative electrodes is illustrated in Scheme 5 which is a two‐electron transfer process.

**Scheme 6:**
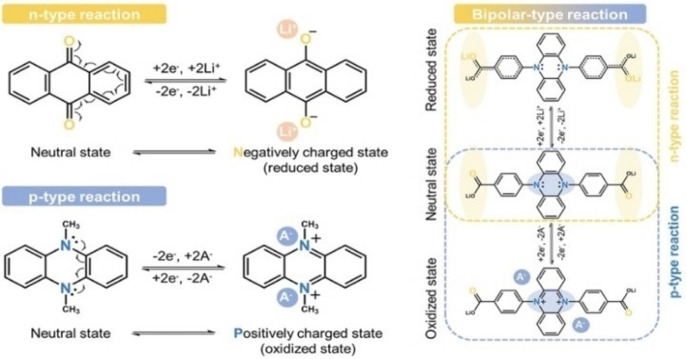
Mechanism of redox reaction occurring at the electrodes for organic supercapacitors. Reproduced from Ref. [166]. Copyright (2022), with permission from John Wiley and Sons.

Another concept behind the storage mechanism in such electrode material can be explained in terms of donor‐acceptor electron exchange. The backbone of charge transfer in most organic compounds is this donor‐acceptor covalent bonding. Before going into the details of the donor‐acceptor level it is important to see the types available to us. We have three types of molecular states in organic compounds viz. n‐type, p‐type, and bipolar‐type.^[166]^ Now, these states can be present in different materials. Then redox reactions will be performed between the materials and the electrolyte ions. But if the material is designed such that both types of the functional group are present in a single material, then both donor states and acceptor states exchange electrons through the material itself and contribute to charge storing. Thus, the donor‐acceptor coupling helps the ion storage in a material. A Mechanism of redox reaction occurring at the electrodes for organic supercapacitor is shown in Scheme 6.

## 3 Electrolytes for Organic Supercapacitor

As one of the integral parts of supercapacitors we now move our discussion to organic electrolytes and the types of such electrolytes used in organic supercapacitors. The organic electrolyte can be classified as a liquid organic electrolyte, organic ionic liquid, and organic polymer electrolyte. The performance of a device is controlled by electrolytes with an effect on the potential window, energy density, power density, cycle life, and the value of specific capacitance. Additionally, other factors like ion size and conductivity of electrolytes can affect the device′s performance by a huge margin. The disadvantages of organic electrolytes are low conductivity, use of volatile and flammable solvents, high‐cost materials, tedious device assembly, difficulty in handling, toxicity, and low‐temperature sustainability. Commonly, the salt used to make organic electrolytes consists of small size anions and large size cations. The conductivity of organic electrolytes is generally found to be low as compared to the aqueous electrolyte. Therefore, the device tends to show lower specific capacitance making the power density less. But as we move towards the organic solvent as the host of electrolytes the stability window increases hence, offering a higher potential window. This enhances the overall energy density of the device compared to the aqueous devices. Another drawback for such electrolytes is the slow kinetics of the ions in the electrolyte solution which results in low specific capacitance. This also causes low power density for these devices. In this current section considering all these advantages and disadvantages, we will discuss the previous research and scope of various organic electrolytes.

### 3.1 Liquid Organic Electrolyte

Liquid organic electrolytes are the basic form of organic electrolytes in which conductive salt is added to the organic solvent. The use of organic electrolytes in conventional SCs is very high because of their large working potential window compared to aqueous electrolytes. An increase in working potential automatically increases the energy density. Generally, the potential range of a device fabricated using liquid organic electrolytes is 2.5 to 3.0 V. One of the most used commercial organic electrolytes is tetraethylammonium tetrafluoroborate (TEABF_4_). Common solvents used are acetonitrile (AN) or propylene carbonate (PC) which usually can sustain an operating window of up to 2.8 V. The performance of SCs depends on factors such as the conductivity of salts, organic solvents, and the size of the ions in action.^[167]^ Azaïs et al. studied the stability of organic electrolytes extensively over the cycle of supercapacitors.^[168]^ They utilized 1 mol L^−1^ of organic electrolyte tetraethylammonium in acetonitrile to achieve a voltage of 2.5 V. Studies showed that the capacity decreases due to the decomposition of the organic electrolyte and this also increased the device resistance. There are other reports where redox additive to the organic electrolyte improved the electrolyte performance. Such work had been reported by Yu et al. where P‐phenylenediamine was added to lithium perchlorate in acetonitrile electrolyte. This strategy gave a high specific capacitance of 68.59 F g^−1^ with a high energy density of 54 Wh kg^−1^.^[169]^ The higher electrochemical performance can be attributed to the fast‐electronic migration and quick faradaic redox reactions. Aslan et al. used 1 M TEABF_4_ in propylene carbonate as an electrolyte with an activated carbon electrode to achieve 112 F g^−1^ specific capacitance.^[170]^ Many other reports can also be seen where organic electrolytes are used as the alternative to increase the potential window of devices whose active electrodes are inorganic materials.[^171^, ^172^, ^173^]

### 3.2 Organic Ionic liquid

Ionic liquids are molten salts in low temperatures below 100 °C. Big asymmetrical organic cations and an organic anion make the whole body of the ionic liquid.^[174]^ Mostly aprotic ionic liquids are used as supercapacitors.[^175^, ^176^] These electrolytes are stable at higher potential and can provide 2.5 to 4 V of device output voltage. Yoshii et al. used the ionic liquid to construct the device‘s highest voltage of 6 V in a battery.^[177]^ The primary advantages of ionic liquid are low volatility, less flammability, and high thermal stability. However, they show certain disadvantages like high cost and high viscosity causing low conductivity. There are many recent reports based on the use of ionic liquid electrolytes mostly in batteries or inorganic electrode‐based supercapacitors.[^178^, ^179^, ^180^] Here we will mention a few reports on the organic electrode‐based supercapacitors using ionic liquids. Arbizzani et al. used galvanostatic polymerization to construct high performing supercapacitor with poly(3‐methylthiophene) as electrode material in 1‐ethyl‐3‐methyl‐imidazolium bis (trifluoromethanesulfonyl) imide (EMITFSI) ionic liquid which was able to work in high temperature.^[178]^ Their device delivered a specific capacitance of 250 F g^−1^. Balducci et al. fabricated a hybrid supercapacitor using activated carbon as the electrode and poly(3‐methylthiophene) with N‐butyl‐N‐methylpyrrolidinium bis(trifluoromethanesulfonyl)imide as the ionic liquid electrolyte.^[179]^ The device showed high cycle stability over 15000 cycles. Cebeci et al. used a novel strategy of using EDOT‐nonylbithiazole‐EDOT based comonomer as electrode materials to achieve charge storage of 5 C cm^−2^ and areal specific capacitance ∼340 mF cm^−2^.^[180]^ The device demonstrated a very linear discharge profile showing good capacitive nature. Wang et al. used polyaniline nanowire arrays with lithium bis(trifluoromethane sulfonyl) (LiTFSI) ionic liquid electrolyte.^[181]^ Here the device successfully delivered ∼950 F g^−1^ specific capacitance, maintaining a high rate capability of 82 % even at a high discharge current of 40 A g^−1^. Tang et al. utilized cationic poly(3,4‐ethylenedioxythiophene)‐DNA composite through in‐situ chemical oxidative polymerization of EDOT‐N monomer in the presence of salmon DNA as a template.^[182]^ They got a maximum specific capacitance of 32 F g^−1^ at 1 mV s^−1^. There are other works where carbon‐based electrodes and metal oxides were employed in the fabricated device along with an ionic liquid as an electrolyte, but those are not within the scope of this review.[^183^, ^184^, ^185^]

### 3.3 Organic Polymer Electrolyte

To avoid leakage in the organic supercapacitor, polymer electrolytes were introduced. These electrolytes are also helpful to prevent the device from corrosion and self‐discharging. Here a polymer matrix is used to hold the electrolyte. These can be classified into two subsections viz. gel polymer organic electrolyte and solid polymer organic electrolyte. The gel polymer electrolyte helps to fabricate flexible SCs which are further modified into stretchable and self‐healing supercapacitors.^[186]^ Holding capacity of the electrolyte makes these devices highly cyclable with convincing long‐term use. Na et al. synthesized a high‐performance fire‐retardant gel polymer electrolyte with a facile epoxy open‐ring polymerization strategy.^[187]^ They used a functional poly(ethylene glycol) copolymer cross‐linking which is a novel flame retardant, with a brominated epoxy resin, and tetrabromo bisphenol as a matrix. With this device, they achieved a high specific capacitance of 166.69 F g^−1^ at 0.5 A g^−1^ current density and a large energy density of 42.19 Wh kg^−1^. Zhu et al. strategized coherent integration of organic gel polymer electrolyte and ambipolar polyoxometalate hybrid nanocomposite electrode.^[188]^ Poly(vinylidene fluoride‐co‐hexafluoropropylene) (PVDF‐HFP) was used as the polymer backbone to host the electrolyte. They achieved ∼40 % increment in the volumetric capacitance using this strategy. Yan et al. played with the hydrogen bonds to improve the ionic conductivity of such materials. They made a hydrogen bond interpenetrated agarose/PVA network to reduce the crystallinity of PVA which provided a higher ionic conductive and also flame‐retardant gel polymer electrolyte.^[189]^ Hillier et al. used green solvent DMSO with TEABF_4_ as salt and polyacrylamide as a polymer to have long‐term performance with 48 % capacity retention after two months.^[190]^

In general, the organic electrolytes were able to provide broad potential windows, better conductivity with few modifications, higher temperature sustainability, high stability, cost‐efficient, and most importantly enviro‐friendly and composed of earth‐abundant molecules.

## 4 Separator: A Highly Rated Component That Can Change the Performance Drastically

As discussed earlier, a separator is also an important part of the device, and now we will focus on the ongoing research direction for various separators used in supercapacitors. Over the years the advancement in flexible and miniaturized devices enables the growth of the separator which provides free ionic flow and keeps the two electrodes separated protecting them from short circuits. Separators can be formed of glassy fiber, aqua gel, polyolefins, rubber, etc.^[191]^ However, here we will focus on the material belonging to the organic family.

### 4.1 Pure Organic Compound‐Based Separator

Most common macro porous separators are made from poly(vinylidene fluoride) (PVDF) and poly(vinylidene fluoride‐co‐hexafluoropropylene) (PVDF‐HFP).^[192]^ These separators show good affinity with the organic liquid electrolyte, high ionic conductivity, and electrolyte soaking due to the porous structure. Macro porosity makes these separators more stable in structure compared to the dense membrane.^[193]^ In recent times the most common separators being used are Nafion and sulfonated poly(ether ether ketone) based membranes.[^38^, ^194^, ^195^, ^196^] The main component of Nafion is a hydrophobic Teflon backbone with hydrophilic sulfonic acid groups attached to it.[^191^, ^197^, ^198^]

### 4.2 Cellulose‐Based Membrane Separator

Another important separator membrane is the cellulose base separator which is abundantly available in nature. Cellulose is a polysaccharide generally found in cotton, wood, bamboo, etc.^[199]^ Main advantages of these materials are low cost, high flexibility, mechanical endurance, thermal stability, good wettability, and the presence of a surface hydroxyl group.^[200]^ Most importantly when we are talking about the flexible supercapacitor, people are more focused on such organic cellulose‐based materials as a separator. Zhao et al. showed how organic bio‐degradable material can be used as the separator with improved performance for the devices.^[201]^ They used a flexible, transparent, and renewable mesoporous cellulose membrane having uniform mesopores of ∼24.7 nm and high porosity of 71.78 %. They used a very simple facile solution‐processed strategy to fabricate the separator. With KOH‐saturated membrane as a polymer electrolyte, they have demonstrated high electrolyte retention of 451.2 wt%, a high ionic conductivity of 0.325 S cm^−1^, and excellent mechanical flexibility and robustness. Torvinen et al. used a low‐cost pigment‐cellulose nanofibril composite for the separator.^[202]^ With optimized drying and wet processing, they improved the flexibility of the separator membrane. Moreover, they worked on standardizing the concentration of the cellulose in the solution during casting. With a 7 % concentration of cellulose, they created a separator that was able to show a higher porosity of 74.90 % and electrolyte uptake of 323.68 %. Even the pore size in this case was improved to a smaller pore size with good uniformity. Another type of cellulose‐based separator which attracts researchers recently is bacterial cellulose.^[203]^ This type of separator has some unique properties i. e. three‐dimensional multilayer network structure and excellent mechanical strength.^[204]^ This is one of the eco‐friendliest separators used in organic supercapacitors. Jiang et al. fabricated a bacterial cellulose separator that possessed a unique fibrous and cross‐linked three‐dimensional network structure for Li‐ion batteries.^[204]^ Similar work was done by Lv et al. where they used an asymmetric all‐solid‐state flexible supercapacitor derived from bacterial cellulose.^[205]^ Using PVA/H_2_SO_4_ as the electrolyte they achieved a flexible supercapacitor with very high capacity and low solution resistance.

### 4.3 Improved Redox Active Separator

So far, we have been talking about the MOF‐based supercapacitor where these devices have excelled in their respective roles as good electrode performers. These materials are being considered the next‐generation electrode materials for supercapacitors because of their high porosity and surface area, uniform cavities and pores, and good thermostability.[^206^, ^207^, ^208^, ^209^] Considering the usefulness of these materials, various reports have been published using MOFs as separator materials.[^155^, ^210^, ^211^] Cai et al. used MOF‐derived conductive carbon nitrides as a separator for flexible supercapacitors.^[210]^ Suriyakumar et al. utilized a MOF‐based separator in batteries which also showed a new direction to the organic materials for supercapacitors.^[197]^ There is another strategy of the redox‐active separator where gel polymer electrodes play the hybrid role of electrolyte as well as separator.^[212]^ In these electrolytes, redox‐active elements like hydroquinone, sulfonic acid, bromic acid, etc show redox contribution to a device. Feng et al. used toughened redox‐active hydrogel as a flexible electrolyte which also acted as a separator showing a conductivity ∼28.5 mS cm^−1^.^[213]^ Aidoudi et al. showed the use of novel hydroquinone sulfonate‐based redox active ionic liquid which can work in the temperature range of 20 to 70 °C.^[214]^ Similar strategies have been employed in batteries also. Pan et al. used a polydopamine‐based redox‐active separator in Li‐ion batteries to improve the performance of the device.^[215]^

## 5 Challenges for Future Advancement

The ongoing research and development in the field force us to understand the challenges behind the organic supercapacitors not progressing as expected to compete in the current energy storage market. We have seen a lot of progress in organic LED, organic transistors, etc. But the main problem associated with the organic compound to be realized in the supercapacitor is the stability of the material when used as the electrode. If we fix the issue with the stability of the material in presence of an electrolyte, the conductivity of the electrode material, stable potential window in combination with a particular electrolyte, capacity retention, cycling stability, etc., organic material can be evolved as a greener and cheaper alternative to the other materials available as the electrode. In the subsequent section, we will highlight the areas where organic supercapacitor faces challenges and the future possibilities of this industry.

### 5.1 Stability of the Material

When we are talking about the stability of organic electrodes, it is very important to take care of the stability of a material. Organic materials can be decomposed on their own. So, the main need for a stable device is stable electrode materials. Two important parameters that can change the state of the organic material are water and oxygen. Water can work in direct form or the vapour state. Over the years water and oxygen have been the bottleneck of their further development. The residual and penetrative water and oxygen in electrochemical cells form electron traps which trigger irreversible side reactions.^[216]^ This becomes a detrimental fact for such supercapacitors electrode for long‐term stability. Zhao et al. utilized a trap‐filling strategy for organic electrode material to have long‐term stability. Two electrode materials BthCz and AQCz, with the lowest unoccupied molecular orbital levels above or near the electron traps (−3.6 to −3.8 eV) exhibit conspicuous stability increments of 68.6 and 26.3 %, respectively, with the optimized DMC concentration of 5×10^−4^ M in acetonitrile electrolyte.^[216]^ If the material is photoactive, sometime light‐induced degradation of the material can also be seen. Small molecular organic electrode materials enjoy favourable high capacity and low cost, but these materials suffer from poor cycling stability and low Coulombic efficiency due to the unavoidable dissolution in aprotic electrolytes which showed the stability of electrodes is a necessity to have industrial applications.^[217]^ Cai et al. implemented the strategy of using high‐concentration electrolytes. They used two dianhydride molecules, namely 1,4,5,8‐naphthalenetetracarboxylic dianhydride (NTCDA) and 3,4,9,10‐perylenetetracarboxylic dianhydride (PTCDA) which help them achieving cycling stability.^[217]^

### 5.2 Stability of the Electrolyte

The next most important issue that we need to encounter is the stability of electrolytes that will provide a higher stable potential window. Most of the organic materials used for an electronic devices are sensitive to water. Again, the basic electrolyte that is used for a supercapacitor is the aqueous electrolyte. This limits the potential window of the device to 1.23 V as beyond the potential electrolyte will decompose into H_2_ and O_2_. Now the question that arises immediately is what can be the alternative to this. A new next‐generation approach to the organic supercapacitor is the use of organic electrolytes. Liquid organic‐solvent‐based electrolytes are the most common and investigated. These electrolytes consist of a liquid organic solvent that accommodates the conductive salt dissolved in it. As mentioned earlier, the electrolytes need to have good electrical conductivity and chemical inactivity towards each of the electrode materials used along with oxidative and reductive stability. The solvent should have high dielectric constant, suitable solubility of the electrolyte salt, and low viscosity. As we are aiming toward green chemistry by using the organic approach, all the components used should be non‐toxic and hence environmentally friendly.

### 5.3 Conductivity of the Material

One of the biggest drawbacks of organic materials is their lower conductivity. Organic compounds do not have free‐charge carriers to conduct electricity. These structures depend on the highest occupied molecular orbital (HOMO) and lowest unoccupied molecular orbital (LUMO) band separation for the electron conductions.^[218]^ The splitting of the HOMO and LUMO level resulting from the interaction of adjacent chains enhance the hole (electron) transportation. To improve the conductivity of these materials many strategies have been employed. One such strategy is the use of electroactive macrocycle building blocks in making functional two‐dimensional porous organic frameworks. Xu et al. strategized the use of conjugated macrocycles that organize into two‐dimensional porous sheets via non‐covalent van der Waals interactions.^[219]^ Molecular structures also help to improve conductivity. One such study was demonstrated by Carlton et al. where they explained that the π‐orbital overlapping helps in conduction. In addition, the degree of overlapping along the conjugated chains defines the degree of electrical conduction.^[220]^

### 5.4 Redox Activity of the Material

**Scheme 7:**
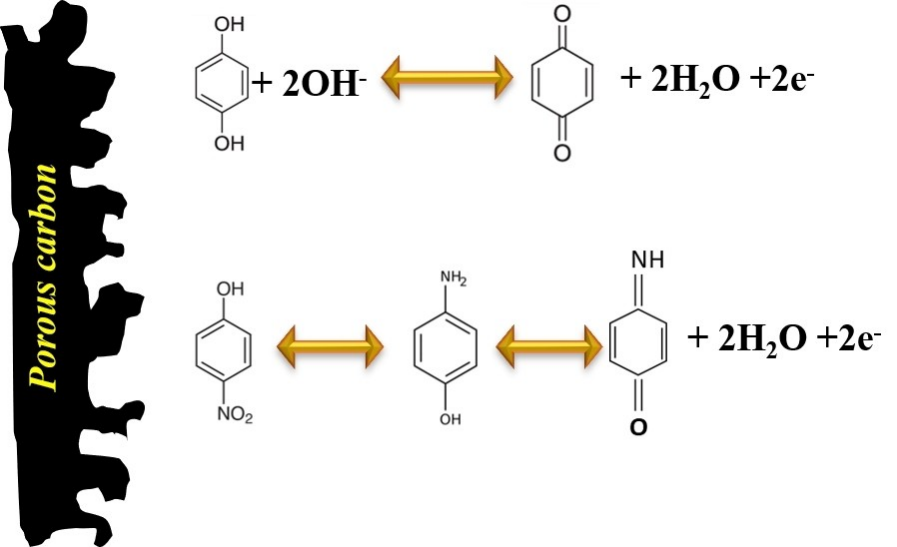
Redox mechanism in organic materials.

Over the years carbon has become the backbone of all the supercapacitors whose working is based on the EDL storage mechanism. In the recent past, much research was done to incorporate the redox mechanism into carbon structures. Carbon nanotubes and graphene sheets have been fictionalized with redox‐active organic functional groups.[^221^, ^222^, ^223^, ^224^] But to achieve a high‐performing pure organic supercapacitor with better redox activity we need organic compounds to be factionalized with redox‐active groups. In recent studies, most of the redox activity comes from the quinone, aminophenol, and amine groups.[^225^, ^226^] The detailed redox reaction mechanism is shown in Scheme 7.

### 5.5 Capacity Retention

Organic materials‐based electrodes show huge promise but over the cycling, the capacity gradually reduces. The main reason is hidden in the material stability as discussed earlier. Along with the reactivity some irreversible reactions are also found which reduces the redox contribution in the electrode material. Thus, the device performance over the cycles reduces.

### 5.6 Carbon Footprint

Another important drawback of using organic elements as an electrode material or separator is the total carbon footprint from a material, as the backbone of organic material is carbon. In both cases, there is a possibility of an increased carbon footprint in making such devices.[^227^, ^228^] But these can be well maintained by calculation of performance obtained from the electrode material and effect cost to produce the material. Also, there is a need to consider the toxicity coming from the other transition metal‐based oxides and sulphides. There come the advantages of such materials over the others.

A comparison of the performance and important properties of inorganic materials has been tabulated in Table 2 for a better understanding of the possible opportunities where organic materials can be found for better energy storage devices. A detailed discussion has been added in the subsequent section describing the opportunities.

**Table 2:** Comparison of advantages and disadvantages in inorganic and organic supercapacitors.

Property	For components used in inorganic supercapacitor	For components used in organic supercapacitor
Conductivity	Relatively very high, some shows very exceptional conductivity	Low, need the incorporation of conducting material blending
Self‐discharge	Moderate to high, the main reason is the use of aqueous electrolyte	Low when ionic liquid is used as a component, Some materials with a higher number of the functional group can
Voltage	Low to moderate depending on the stability of the electrolyte	Very as most of them use organic electrolytes which better stability window compared to 1.23 V for water
Capacity	Very High	Low to moderate values observed till now
Energy density and power density	Very high‐power density but the low energy density	Low power density but the high energy density
Kinetics	Fast kinetics are observed	Low kinetics for ion flow ad redox reactions observed
Thermal stability	Mostly stable under higher temperatures	Thermal stability is low except for conducting polymers
Solubility in the case of electrolyte	Don′t withstand a high potential window and also most of them show redox behaviour within a lower potential region	Can easily provide 2–4 V od stable potential window
Preparation complexity	To have good morphologies need to employ complex and multi‐step synthesis	Most of them are synthesizable by simple to moderate strategies
Long term stability	Highly stable	Low to moderate
Environfriedlyness	Most of the inorganic materials are highly toxic to the environment	Good towards environment and carbon footprint is less and some materials can easily decompose to the environment

## 6 Opportunities in This Field with the Future Direction

After seeing the progress in this field, we can now discuss the direction in where we can progress to improve the performance further. Current progress in the field is to focus on the materials which can be utilized both in the supercapacitor and batteries. Most importantly combining the working principle of both types of devices to make hybrid devices. Here comes the advantage of organic materials as the working electrode. These materials can well behave as hybrid electrodes. Already the metal‐organic framework (MOF) has established that it can be a good alternative to the metal oxide‐based supercapacitor. The following advantages of these materials make them the future material for next‐generation supercapacitors.

### 6.1 Abundancy of Material

First of all, for any material used as an electrode for a commercial supercapacitor, the availability of the material or the cost of synthesis are the primary things that need to be considered. The main constituent elements for the organic electrode materials used in supercapacitors are C, H, N, O, S, etc. These are the most abundant light elements present on the earth. These materials are generally present in seawater, pedosphere, or directly in the bioresources available on earth. So, their demand and supply will be better in comparison to lithium‐based devices.

### 6.2 Synthesis of Material and Availability in the Direct Form

Some of the organic materials can easily procure from the petroleum industry as byproducts.[^229^, ^230^] A small fraction of crude oil is used to synthesize olefins and aromatic compounds which can be used as the organic electrode materials for supercapacitor devices. The other positive factor behind these materials is that whenever bioresources are utilized, the problem of cost and availability automatically reduces. If we try to synthesize these materials, they can be easily synthesizable, and the temperature utilized during their making is room temperature or 150 °C.

### 6.3 Diversity of the Material

When we are talking about inorganic materials, there is a limited number of materials and we look forward to the strategy to modify the morphology for achieving high performance. For organic electrodes, the material can be designed in diverse forms concerning both the structures and electrochemical properties. Now the future is also dependent on flexible and integrable devices. The organic device can be moulded easily as a flexible device and also smoothly integrable into the wearable device.

### 6.4 Recyclability and Environmental Friendliness

The next important aspect behind these materials is the easy recycling to reuse them as fuels after completion of their utilization as electrodes/electrolytes. With the increase in environmental concern, we are moving forward with materials that have fewer effects on environmental damage. These materials are proven to be good for the environment.

### 6.5 Achieving Performance with Safety

The last but the most attractive one among the advantages or opportunities behind such devices is the potential window that we can play with. There are reports where 6 V of potential window can be easily obtained from such devices. The aqueous‐based devices have the drawback of the lower potential window with the possibility of device blast due to the uncontrolled water splitting within it. But for organic devices, the potential is much higher and safety issues can also be easily handled. So, these devices automatically prove to be stable ones with high energy and power densities. The main aim of these devices is cycling stability for long‐term usability.

## 7 Conclusion and Perspective

The article has summarized the recent advancement in the field of organic supercapacitors. There has been a paradigm shift in the appearance of articles on organic supercapacitors. Be it material chemistry or the construction of the device, an organic supercapacitor is gaining popularity day by day due to its many attractive properties. Many of the early challenges in the development of the organic supercapacitor have been mitigated over the years. From the electrode to the separator, all the components have been tuned to get better and environment‐friendly devices. The stability issue is the most tolerant factor for an organic supercapacitor and a lot of new materials with better stability have been addressed in many recent works of literature. Ranging from the organic polymer to polymer‐polymer composite, the functionalization of organic chains with redox active group, introducing heteroatom or atomic group has paved the path for better electrochemical devices. The newer concepts of molecular contortion of the material have been employed to get high‐performing supercapacitors. Not only that, the cycling stability is not compromised to achieve better performance. Recent development in overcoming the challenges shows that significant progress has been made to realize eco‐friendly organic supercapacitors. Further work is needed to maintain the pace of progressing newer and more cost‐efficient devices. We have also summarized the mechanism utilized by these next‐generation organic‐based devices.

Almost over two centuries lot of investment has been done in the field of energy storage. Starting from the basic carbon‐based devices to the evolvement of the transition metal‐based supercapacitors has been gone through many changes and improvements in material morphology or chemistry of the electrode and electrolyte. In the 21^st^ century, most supercapacitors are controlled by reversible redox chemistry. But the next generation way forward for energy storage systems is organic‐based devices. We have already seen a paradigm shift in organic electronics. Considering the global need for renewable energy we should focus on energy storage devices based on organic substances.

To conclude, though we are in the early stage of organic material‐based supercapacitors, it is in the near future when with the advancement of redox‐active organic compounds and choices of materials we can get better and more sustainable organic devices. Till now we have just arrived at a few possibilities. We hope with this comprehensive review will be a source of original research and will open many dynamic new dimensions to fresh ideas for future generation readers.

## Conflict of interest

The authors declare no conflict of interest.

8

## Biographical Information

*Sudipta Biswas is a postdoctoral researcher at the Ben Gurion University of the Negev. He obtained his Ph.D. in 2021 from the Indian Institute of Technology Kharagpur, India, and worked on sodium‐ion‐based storage systems for e‐vehicles. He is an experienced researcher in the field of supercapacitors*.

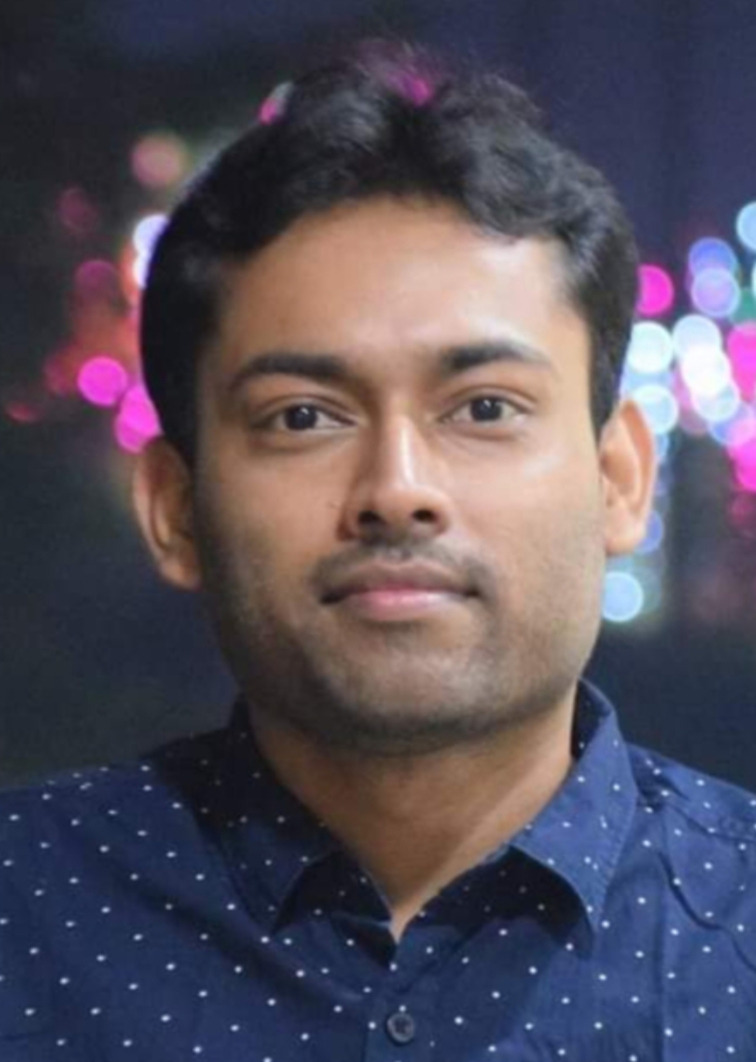

## Biographical Information

*Ananya Chowdhury is a postdoctoral researcher at the Indian Institute of Technology Bombay. She obtained her Ph.D. in 2021 from the Indian Institute of Technology Kharagpur, India, and worked on sodium‐ion‐based storage systems. She is an experienced researcher in the field of supercapacitors and materials synthesis*.

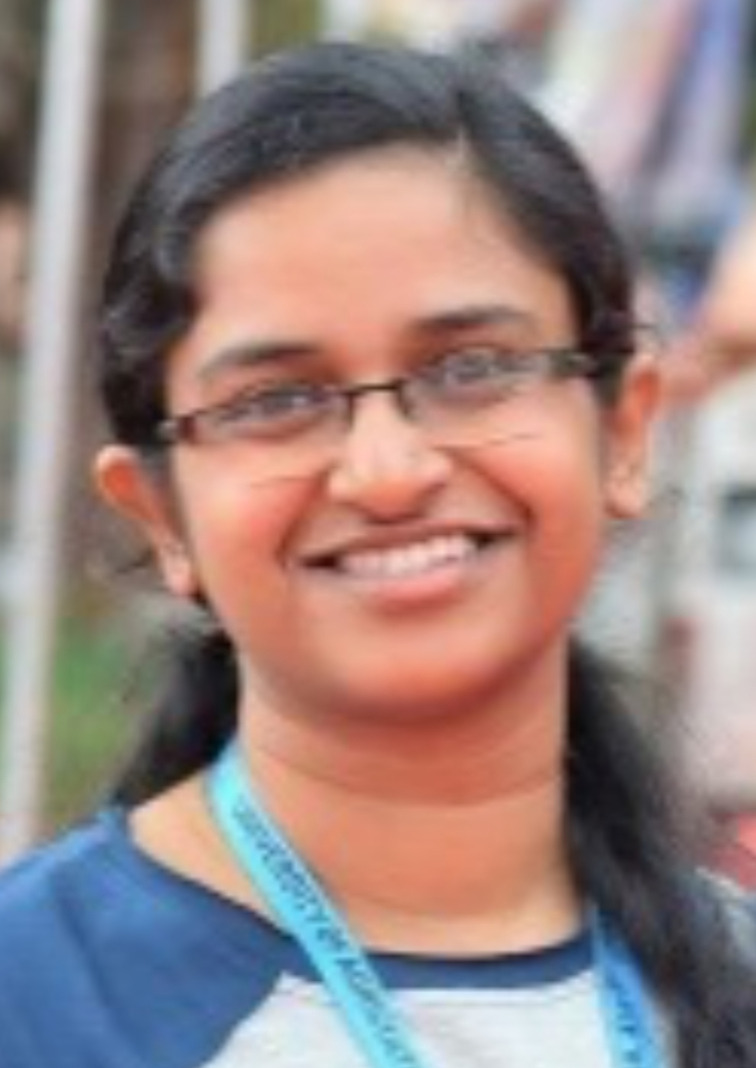

## Data Availability

Data sharing is not applicable to this article as no new data were created or analyzed in this study.
